# Deep Medullary Vein and MRI Markers Were Related to Cerebral Hemorrhage Subtypes

**DOI:** 10.3390/brainsci13091315

**Published:** 2023-09-13

**Authors:** Dan Wang, Yayun Xiang, Yuling Peng, Peng Zeng, Bang Zeng, Ying Chai, Yongmei Li

**Affiliations:** 1 Department of Radiology, The First Affiliated Hospital of Chongqing Medical University, 1# Youyi Road, Yuan Jiagang, Chongqing 400010, China; 2 Department of Radiology, Mianyang Central Hospital, 12# Changjia Lane, Mianyang 621000, China; 3 Department of Radiology, People’s Hospital of Shapingba District, 44# Xiaolongkan New Street, Chongqing 400010, China

**Keywords:** deep medullary vein, MRI markers, intracerebral hemorrhage, cerebral small vessel disease

## Abstract

Background: To explore the performance of deep medullary vein (DMV) and magnetic resonance imaging (MRI) markers in different intracerebral hemorrhage (ICH) subtypes in patients with cerebral small vessel disease (CSVD). Methods: In total, 232 cases of CSVD with ICH were included in this study. The clinical and image data were retrospectively analyzed. Patients were divided into hypertensive arteriopathy (HTNA)-related ICH, cerebral amyloid angiopathy (CAA)-related ICH, and mixed ICH groups. The DMV score was determined in the cerebral hemisphere contralateral to the ICH. Results: The DMV score was different between the HTNA-related and mixed ICH groups (*p* < 0.01). The MRI markers and CSVD burden score were significant among the ICH groups (*p* < 0.05). Compared to mixed ICH, HTNA-related ICH diagnosis was associated with higher deep white matter hyperintensity (DWMH) (OR: 0.452, 95% CI: 0.253–0.809, *p* < 0.05) and high-degree perivascular space (PVS) (OR: 0.633, 95% CI: 0.416–0.963, *p* < 0.05), and CAA-related ICH diagnosis was associated with increased age (OR: 1.074; 95% CI: 1.028–1.122, *p* = 0.001). The DMV score correlated with cerebral microbleed (CMB), PVS, DWMH, periventricular white matter hyperintensity (PWMH), and CSVD burden score (*p* < 0.05) but not with lacuna (*p* > 0.05). Age was an independent risk factor for the severity of DMV score (OR: 1.052; 95% CI: 0.026–0.076, *p* < 0.001). Conclusion: DMV scores, CSVD markers, and CSVD burden scores were associated with different subtypes of ICH. In addition, DMV scores were associated with the severity of CSVD and CSVD markers.

## 1. Introduction

Cerebral small vascular disease (CSVD) includes a series of pathological, imaging, and clinical processes that affect the cerebral arterioles, venules, and capillaries [1,2]. It is the most common etiology for primary intracerebral hemorrhage (ICH). Intracerebral hemorrhage is among the most severe forms of acute stroke and is a serious neurological crisis [3,4], and it is associated with high mortality and morbidity. To date, the mortality rate has been greatly reduced due to the improvement of technology for treating cerebral hemorrhage. However, survivors of cerebral hemorrhage are often accompanied by many neurological sequelae that impair quality of life. In clinical treatment, it is necessary to distinguish ICH subtypes in patients with ICH, as the risk of recurrence of cerebral amyloid angiopathy (CAA)-related ICH is approximately 7% per year [5], and the risk of recurrence of non-CAA-related ICH is significantly lower. Moreover, the differentiation of cerebral hemorrhage subtypes is also meaningful for later clinical decision-making.

The image markers of CSVD include white matter hyperintensity (WMH), cerebral microbleed (CMB), enlarged perivascular space (PVS), and lacune [6,7]. The total CSVD burden score combines these neuroimaging variables and reflects the severity of CSVD. However, it does not directly reflect the pathological development of CSVD at the level of small vessels. In patients with CSVD, microvascular lesions occurred before the appearance of MRI neuroimaging markers. Previous studies [8,9] on CSVD have mainly focused on cerebral arterioles, whereas relatively few studies have focused on veins. However, veins play an important role in the pathological process of CSVD. The deep medullary vein (DMV) is a component of the cerebral medullary vein system, which can be imaged on Susceptibility Weighted Imaging (SWI) [10]. Its main function is to drain the blood flow of the white matter to the subependymal region [11] and then drain the blood flow into the deep venous system. At present, there are no studies to investigate the manifestations of DMV in patients with different ICH subtypes. In addition, previous studies [12,13] have explored the relationship between DMV and neuroimaging indicators in patients with CSVD with ischemic infarction. However, in patients with CSVD with ICH, the relationship between DMV and neuroimaging indicators and the relationship between DMV and the severity of CSVD has not been widely studied.

Therefore, we investigated the differences in the performance of DMV and CSVD markers in different subtypes of ICH, hoping to provide some information for the clinical classification of ICH. In addition, we also investigated the relationship between DMV and CSVD markers and the CSVD burden score in patients with ICH.

## 2. Methods and Materials

### 2.1. Patients

This study has a cross-sectional, retrospective, and single-institutional design. This study was approved by the institutional review boards of our hospital, with individual consent waived due to the retrospective nature of the study. The study subjects were consecutive patients admitted to the First Affiliated Hospital of Chongqing Medical University with a diagnosis of spontaneous ICH. Clinical and image data from CSVD patients from January 2014 to November 2022 were retrospectively collected. The clinical data included sex, age, hypertension, tobacco use, alcohol use, diabetes, hyperlipidemia, coronary heart disease, and stroke history. Diabetes was defined as random blood glucose > 11.1 mmol/L, glycated hemoglobin > 6.5 mmol/L, or current use of anti-diabetic medications. Hypertension was defined as systolic blood pressure ≥ 140 mmHg, diastolic blood pressure ≥ 90 mmHg, or treatment with antihypertensive drugs. Dyslipidemia was defined as a total serum cholesterol level ≥ 240 mg/dL, low-density lipoprotein cholesterol level of ≥160 mg/dL, or current use of lipid-lowering drugs after being diagnosed with dyslipidemia. Tobacco use and alcohol use were defined as continued smoking or drinking to the present. The coronary heart disease and stroke history were documented in the medical records.

**Figure 1:**
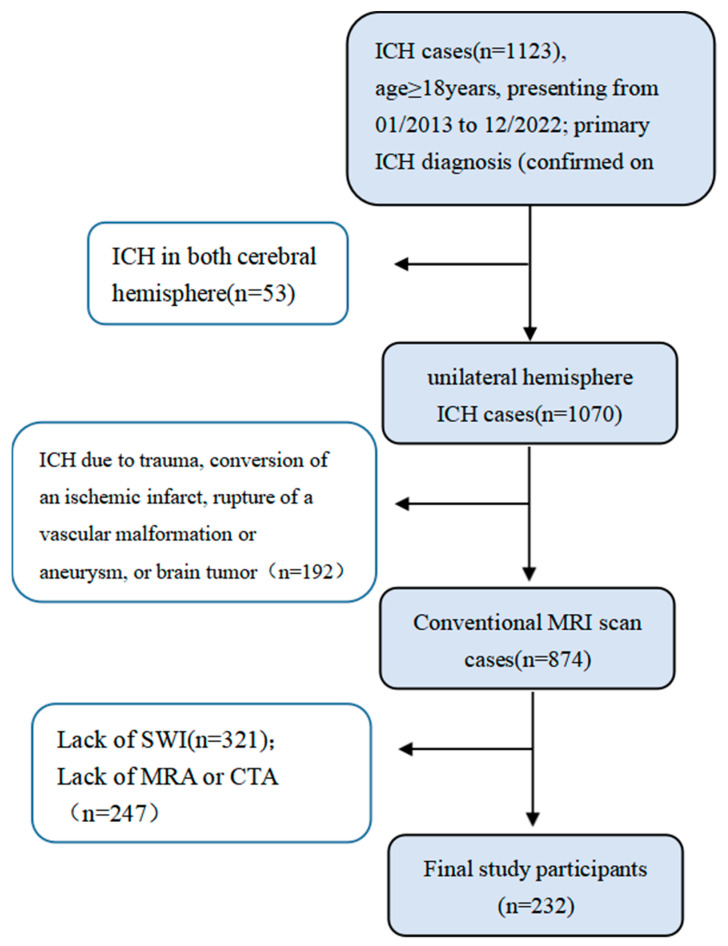
Enrollment flow chart and study inclusion and exclusion criteria.

Inclusion criteria: (1) diagnosed with primary ICH; (2) all participants were over 18 years old at the time of acute primary ICH; and (3) underwent an magnetic resonance imaging (MRI) scan and MR or computer tomography (CT) angiography. Exclusion criteria: (1) ICH in both cerebral hemispheres; (2) ICH caused by trauma, ischemic infarction transformation, vascular malformation, aneurysm rupture, or brain tumor trauma; (3) poor image quality. Figure 1 shows a flow chart representing the enrollment of patients into the study. Baseline information about the patients was collected from the medical records.

### 2.2. Magnetic Resonance Imaging Protocol

All patients underwent multimodel MRI with T1WI, T2WI, T2 FLAIR, and SWI sequences on a 3.0T MR scanner (uMR 790, United Imaging, Shanghai, China), a 3.0T MR scanner (Skyra, Siemens, Erlangen, Germany), or a 3.0T MR scanner (GE Healthcare, Milwaukee, WI, USA). SWI sequence in the uMR 790 scanner with the following parameters: repetition time = 30 ms; echo time = 20 ms; flip angle = 15°; slice thickness = 2 mm; field of view = 230 × 200 mm^2^; and matrix number = 448 × 224. The parameters of SWI for Skyra were: repetition time = 26 ms; echo time = 20 ms; flip angle = 15°; slice thickness = 2 mm; field of view = 230 × 194 mm^2^; and matrix number = 256 × 245. The parameters of SWI for GE were: repetition time = 49 ms; echo time = 40 ms; flip angle = 15°; slice thickness = 2 mm; field of view = 240 × 200 mm^2^; and matrix number = 256 × 224. The rest of the sequence information is in the supplementary material.

### 2.3. MRI Data Analysis and CSVD Score

We evaluated WMH, CMB, PVS, and lacuna. WMH was defined as abnormal hyperintensity of periventricular white matter or deep white matter on T2-FLAIR sequences using the Fazekas scale, in which both deep and periventricular regions ranged from 0 to 3 [14]. Rating score periventricular white matter hyperintensity (PWMH): 0 = No lesions, 1 = Focal lesions, 2 = Beginning confluence of lesions, and 3 = Diffuse involvement of the entire region/with or without involvement of U fibers. Rating score deep white matter hyperintensity (DWMH): 0 = No lesions, 1 = One focal lesion, 2 = More than one focal lesion, and 3 = Confluent lesions. CMB was defined on axial SWI as a hypointense signal with a diameter less than 10 mm, except for calcification and abnormal iron deposits [15]. The location and number of CMBs were recorded. The enlarged PVS was defined on T2WI as small dot-like or linear fluid signals. PVSs of the basal ganglia (BG) were assessed using a validated 4-point visual rating scale (0 = no PVS, 1 ≤ 10 PVS, 2 = 11–20 PVS, 3 = 21–40 PVS, and 4 ≥ 40 PVS) [16]. Lacuna was defined as round or ovoid subcortical lesions between 3 and 15 mm in diameter, and it showed a hypointense signal on T1 images with the corresponding edge hyperintense signal on T2-FLAIR images.

The CSVD burden score ranges from 0 to 6 points [4]. One point: (a) lacuna; (b) 1–4 CMBs; (c) moderate-to-severe BG PVS (PVS > 20); and (d) moderate WMH (periventricular plus deep WMH grade 3–4). Two points: (a) ≥5 CMBs and (b) severe WMH (periventricular plus deep WMH grade 5–6). Intraclass correlation coefficients for 50 random samples were used to assess interobserver agreement.

### 2.4. ICH Subtype

According to the locations of ICH and CMB, patients with ICH are classified as having HTNA-related, CAA-related, or mixed ICH [4]. HTNA-related ICH was defined as severe deep (BG, thalamus, brainstem, or cerebellum) cerebral hemorrhage, regardless of whether there was deep CMB. However, there should be no lobar ICH or CMB. CAA-related ICH was defined as lobar hemorrhage, regardless of whether there was lobar CMB. Meanwhile, these participants were diagnosed with probable CAA by the modified Boston criteria [17]. Finally, mixed ICH had both image features of the previous two types, which were shown as lobar ICH or CMB and deep ICH or CMB.

### 2.5. Deep Medullary Veins Score

**Figure 2:**
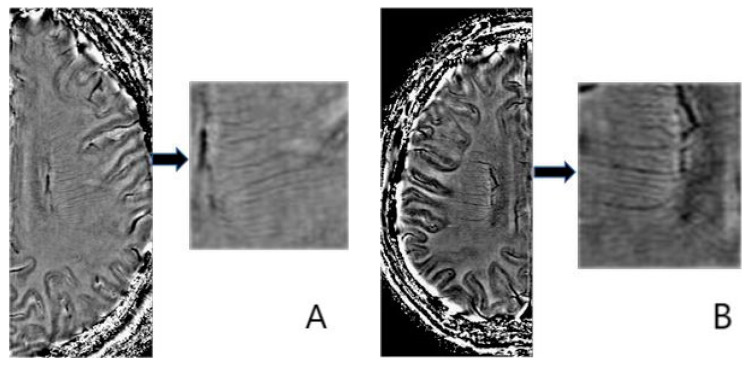

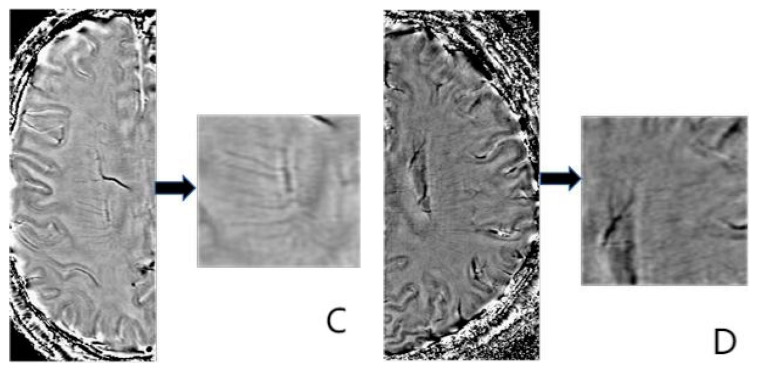
Deep medullary vein scoring system. (**A**) shows score 0 with continuous vein, uniform signal of vein; (**B**) shows score 1 with continuous veins, but one or more veins have uneven signals; (**C**) shows score 2 with one or more veins are discontinuous and with faint visibility; and (**D**) shows score 3 with no continuous veins found.

All images were evaluated by two neuroradiologists who were completely unaware of the patients’ clinical data. The DMV score was based on the SWI sequences. To cover most of the DMV, we selected the region from the level of ventricles directly above the basal nucleus to the level of the disappearance of ventricles. To obtain a more accurate DMV score, we mainly used SWI phase images as the main evaluation method, combined with magnitude images and SWI images for comprehensive evaluation. The DMV was scored based on a previous study [18] and on the evaluation of the observation continuity and visibility of the DMV (Figure 2). To avoid the impact of intracerebral hemorrhage on the DMV score, we scored the DMV on the contralateral cerebral hemisphere of the ICH. Interobserver agreement was assessed using the intraclass correlation coefficient (ICC) of a random sample of 50 individuals.

### 2.6. Statistical Analysis

Normal data are presented as the means ± standard deviations, and analysis of variance was used for comparisons between groups. Nonnormal data are shown as medians and interquartile ranges. The Kruskal-Wallis test was used for comparisons between groups. The chi-square test was used for counting data. First, the clinical and imaging characteristics of ICH subtypes were compared. Then, logistic regression analysis was performed to analyze the relationship between the DMV and individual markers and the DMV and CSVD burden scores. The diagnostic value of DMV for CSVD severity was evaluated by receiver operating characteristic (ROC) curve analysis. SPSS 21.0 statistical software was used for statistical analysis. Statistical significance was defined as *p* < 0.05.

## 3. Result

### 3.1. Subject Characteristics in Different ICH Subtypes

We initially screened 1123 consecutive primary ICH cases enrolled for inclusion in subsequent analyses. After the application of prespecified inclusion and exclusion criteria, 232 consecutive patients were enrolled in the study. Among the excluded patients, missing or inadequate MRI data (SWI) and MRA or CTA were the most important reasons (796/1123). The mean age of the subjects was 61.17 years, ranging from 23 to 89, and 65.9% (153/232) were men. Detailed information about the different ICH subtypes in this study sample is summarized in Table 1. The number of patients with HTNA-related and mixed ICH was similar (96 vs. 98), whereas the number of patients with CAA was the lowest (38). Among the demographic indicators, age, tobacco use, alcohol use and hypertension were statistically significant among the subtypes of ICH. Regarding the MRI data and CSVD burden score, all the variables were statistically significant among the subtypes of ICH except the P-DMV (parietal lobe deep medullary vein) score. An interobserver agreement was assessed using a random sample of 50 individuals. The agreement between observers was excellent for the DMV score of SWI (ICC = 0.906, *p* < 0.001) and for the CSVD burden score on T2-FLAIR images (ICC = 0.953, *p* < 0.001). The ICC for the other variables is shown in Table S2.

### 3.2. Subject Characteristics in Different DMV Groups

According to the DMV score, the patients were divided into three groups. Scores of 0–3 points were the mild group, 4–6 points were the moderate group, and 7–9 points were the severe group. Demographic indicators and MRI data are shown in Table 2. The moderate DMV group accounted for the most patients (143/232, 61.63%). The DMV score of patients increased with increasing age. Among the demographic indicators, age, hypertension, and stroke history were statistically significant. Stroke history was significant in the preliminary statistical analysis (*p* < 0.05) but not in the pairwise comparison (mild vs. severe group). All the MRI variables were statistically significant among the DMV groups except for the lacuna. Among these MRI variables, only two markers, DWMH and CSVD burden score, were significantly different between any two DMV groups.

### 3.3. Relationship between DMV and Other Variables

The relationship between the DMV score and other variables is shown in Table 3. The DMV score was positively correlated with age, DWMH, PWMH, and CSVD burden score (all *p* < 0.001) and positively correlated with hypertension, stroke history, PVS, and CMB (all *p* < 0.05). In the univariate regression analysis, CSVD burden score and age were independent risk factors for the severity of DMV (all *p* < 0.001). Hypertension, PVS, and CMB were also independent risk factors for DMV severity (*p* < 0.05). In multivariate logistic regression analysis, age was an independent risk factor for the severity of DMV (OR: 1.052; 95% CI: 0.026–0.076, *p* < 0.001) after adjusting for other factors.

### 3.4. ROC in DMV Groups

The diagnostic efficacy of age and MRI data in differentiating the DMV score at each stage is shown in Table 4. The AUC value of the mild and severe groups was the highest (AUC = 0.879), and the value was the lowest (AUC = 0.668) in the comparison between the moderate and severe groups. Regarding the MRI data, the AUC value of the CSVD burden score was the highest in the mild–moderate and mild–severe groups’ discrimination, with values of 0.695 and 0.763, respectively. In the comparison of the moderate and severe groups, the AUC value of PWMH was the highest (AUC = 0.628). Sensitivity is the probability of actually having the disease and being diagnosed as positive. Specificity is the probability of not actually having the disease and being diagnosed negative.

### 3.5. Logistic Regression Analysis of ICH Subtypes

For the risk factor analysis of the ICH subtype, we used multivariate logistic regression analysis (Table 5). In univariate analysis, alcohol use and PVS were significantly different in the CAA group compared to the mixed group (all *p* < 0.05). Hypertension, lacuna, DWMH, PWMH, and CSVD burden score were also risk factors (all *p* < 0.001). The CSVD score was a comprehensive evaluation based on these variables (CMB, PVS, lacuna, and WMH). To avoid interactions between them, the CSVD burden score was removed in the multivariate logistic regression analysis. ICH subtypes were based on the location of CMB, so CMB was also removed. In multivariate logistic regression analysis, hypertension and age were significantly different in the CAA group compared to the mixed group (*p* = 0.001). In the CAA-related and mixed ICH groups, patients with increased age were more likely to develop CAA-related than mixed ICH (OR: 1.074; 95% CI: 1.028–1.122, *p* = 0.001), whereas patients with hypertension were less likely to develop CAA (OR: 5.169; 95% CI: 1.969–13.567, *p* = 0.001).

In the HTNA-related and mixed ICH groups, age, PVS, CMB, lacuna, PWMH, DWMH, CSVD score, and DMV score were significantly different. Compared to patients with HTNA-related ICH, patients with mixed ICH were older and had higher WMH, a higher incidence of lacuna, more CMB, high-degree PVS, higher DMV score, and a higher CSVD score (all *p* < 0.05). Compared to mixed ICH, HTNA-related ICH diagnosis was associated with higher DWMH (OR: 0.452, 95% CI: 0.253–0.809, *p* < 0.05) and high-degree PVS (OR: 0.633, 95% CI: 0.416–0.963, *p* < 0.05) in the multivariate analysis. Increased DWMH and PVS were less likely to develop HTNA-related ICH than mixed ICH.

## 4. Discussion

In our study, we found that the demographic variables of age, hypertension, and stroke history were significantly different among the DMV groups. Age, hypertension, and stroke history were positively correlated with DMV scores, and age was an independent risk factor for DMV scores. Age is a major risk factor for CSVD [6], and it can affect DMV. Previous pathological studies have found that vein wall collagen thickening increases with age [12]. Venous collagen hyperplasia can lead to thickening of the vessel wall and narrowing of the lumen, which eventually leads to occlusion of the lumen [19]. With age, increased vascular resistance leads to low blood flow within the brain, which disrupts DMV visibility and leads to an increased DMV score. Similarly, vascular compliance decreased, and vascular resistance increased, leading to an increased incidence of hypertension. This might be the reason why the DMV score was positively correlated with the incidence of hypertension.

In terms of PVS, we observed a significant difference between the mild and severe DMV groups. PVS is positively correlated with DMV scores. This result is consistent with previous studies [20]. The perivascular space has been shown to be the basic structure of the glial–lymphatic pathway, and it is a fluid-filled space surrounding penetrating arteries or veins [21]. The enlarged perivascular space is thought to be a sign of lymphatic fluid deposition [22]. At the same time, due to venous insufficiency, perivenous glial lymphatic drainage channels are destroyed, resulting in fluid retention and enabling PVS imaging.

We also found that DWMH and PWMH were significantly different among the DMV groups, and both were positively correlated with the DMV score. The higher the DMV score, the more serious the WMH around the periventricular and deep areas, which was in line with previous studies [7,13]. Previous histopathological findings showed noninflammatory periventricular venous lesions in WMHs with concentric collagen deposition leading to intramural thickening, narrowing, and, ultimately, venous occlusion [23]. Collagenosis of the DMV is the underlying pathophysiology of leukoaraiosis in CSVD [24]. Some studies have also explored the relationship between DMV and WMH [12,25]. Collagen deposition in the DMV impairs the venous drainage channels, leading to vasogenic edema and thus white matter hyperintensity imaging [12,25]. Meanwhile, in our study, the correlation coefficient between PWMH and DMV scores was smaller than that of DWMH. This result is not consistent with the findings of Zhang et al. [26]. This might be explained by the fact that although the venous blood of deep cerebral white matter is mainly drained by superficial medullary veins, there are communication branches between superficial medullary veins and deep medullary veins. Meanwhile, PWMH assessment may be biased due to the physiological WMH around the lateral ventricle. In addition, PWMH and DWMH were positively correlated with total CSVD burden score.

We found that CMB was significantly different between the mild and severe DMV groups. It is positively correlated with the DMV score. This result is in line with a previous study. That study suggests that DMV disruption is associated with microbleeds, especially non-strict lobar microbleeds and extensive microbleeds [27]. Venous insufficiency can increase venous pressure, cause peripheral interstitial edema, and lead to blood-brain barrier disruption [25], which has been shown to be associated with microbleeds [28]. In terms of lacuna, we did not observe significant differences among DMV groups. However, it has a positive and statistically significant correlation with the DMV. This result is partly in line with some previous studies [7,25,26]. Obstruction of the periventricular veins might lead to an increase in venous pressure, venular dilatation, or venular blood-brain barrier disruption [29]. Disruption of the BBB is thought to be one of the mechanisms leading to lacuna formation. Venous outflow obstruction caused by venous ischemia may be one of the causes of lacunae [30]. Therefore, we believe that DMV is involved in the pathological mechanism of CSVD to some extent.

In our study, the CSVD burden score was significantly different among DMV groups, and it was positively correlated with the DMV score. This result was consistent with some previous results [6,7,13]. As mentioned above, there was a relationship between the DMV score and all four markers of CSVD, so there would also be an association between the DMV score and the SCVD burden score. Meanwhile, the diagnostic efficacy of the CSVD burden score was higher among the DMV groups except for age, especially between the mild–moderate and mild–severe groups. The CSVD burden score was a composite indicator based on four markers, and the DMV score was an indicator to evaluate venules. We believe that the combination of these two factors will provide a more comprehensive assessment of the severity of CSVD.

Between group comparisons, patients with mixed ICH had a more pronounced classic vascular risk factor burden than patients with CAA-ICH and HTNA-related ICH. Hypertension and age were significantly different among the three subtypes of ICH. Compared with the mixed ICH group, age was an independent risk factor in the CAA group, and hypertension was a protective factor in the CAA group. The mixed group showed more similarities with HTNA-related ICH patients regarding hypertension, diabetes, and hyperlipidemia prevalence. This result was consistent with previous research findings [31]. A previous study has shown that patients with mixed hemorrhage have more clinical phenotypic similarities with deep hemorrhage than lobar hemorrhage [31]. DWMH and PVS were higher in patients with mixed ICH than in those with HTNA-related ICH. The diagnostic efficiency of the CSVD burden score was highest in the CAA-related group and HTNA-related group compared with the mixed ICH group, respectively. The DMV score was significant only between the HTNA and mixed ICH groups. We speculated that the reason may be that among the patients with mixed intracerebral hemorrhage in this group, CAA overlaps more with mixed intracerebral hemorrhage but less with HTNA intracerebral hemorrhage. Further research is needed.

We also acknowledge our limitations. First, our study is retrospective and cross-sectional. We did not have a longitudinal follow-up of imaging variables or patients’ cognitive status. Second, the DMV score was measured directly and visually based on a qualitative scoring system. At present, some studies have explored the relationship between DMV and CSVD through the number, length, tortuosity, inhomogeneity, and density of DMV. In addition, we did not evaluate MRI images based on machine learning in this article. In future studies, machine learning will be used to evaluate MRI images to reduce human error. Third, the data collection period (from 2014 to 2022) is very long, so different types of MRI machines collected the data. In future research, we will further explore the above limitations. At the same time, we also have some strengths. First, we analyzed the relationship between the DMV score and other indicators of CSVD. Second, we investigated the DMV score and other indicators of CSVD based on patients with ICH, and most of the previous studies were based on patients with ischemic stroke.

## 5. Conclusions

In conclusion, the DMV score, CSVD markers and CSVD burden score were different among subtypes of ICH. DMV scores were positively correlated with all CSVD markers except lacuna. Therefore, we believe that DMV is also associated with the severity of CSVD. Although, some of the variables in our study have certain significance for the identification of ICH subtypes, which can help clinicians to identify ICH subtypes and clinical management of ICH patients. More research is needed before they can be incorporated into the routine clinical application of identifying ICH.

## Figures and Tables

**Table 1:** Clinical and imaging characteristics and comparisons among ICH subtypes.

Clinical and Imaging Characters	ICH (*n* = 232)			*p*-Value
	CAA-Related (*n* = 38)	HTNA-Related (*n* = 96)	Mixed (*n* = 98)	
Age (years), mean (SD)	65.00 ± 15.61 *	56.45 ± 13.67 ^†^	64.32 ± 11.81	<0.001
Male, *n*(%)	17(44.7) *	73(76.0)	63(64.3) ^#^	0.002
Hypertension, *n*(%)	19(50.0) *	73(76.0)	84(85.7) ^#^	<0.001
Alcohol use, *n*(%)	32(84.2) *	59(61.5)	65(66.3) ^#^	<0.05
Tobacco use, *n*(%)	28(73.7) *	49(51.0)	62(63.3)	<0.05
Diabetes, *n*(%)	5(13.2)	22(22.9)	28(28.6)	0.167
Hyperlipidemia, *n*(%)	5(13.2)	18(18.8)	18(18.4)	0.742
Coronary heart disease, *n*(%)	2(5.3)	1(1.0)	5(5.1)	0.202
Stroke history, *n*(%)	4(10.5)	5(5.2)	11(11.2)	0.280
PVS, median (IQR)	1.5(1, 2)	1(1, 2) ^†^	2(1, 3)	<0.001
CMB, median (IQR)	1(0, 1.25)	1(0, 1) ^†^	2(1, 2) ^#^	<0.05
Lacuna, *n*(%)	12(31.6)	39(40.6) ^†^	69(70.4) ^#^	<0.001
DWMH, median (IQR)	1(0.75, 2)	1(0, 2) ^†^	2(2, 3) ^#^	<0.001
PWMH, median (IQR)	1(0, 2)	1(0, 2) ^†^	2(2, 3) ^#^	<0.001
CSVD score, median (IQR)	2(1, 3)	2(1, 3) ^†^	5(3, 5) ^#^	<0.001
FDMV score, median (IQR)	2(2, 3)	2(2, 2) ^†^	2(2, 3)	0.002
PDMV score, median (IQR)	2(1, 2)	2(1, 2)	2(1, 2)	0.077
ODMV score, median (IQR)	2(1, 2)	2(1, 2) ^†^	2(1, 2)	0.002
TDMV score, median (IQR)	6(5, 6)	5(4, 6) ^†^	6(5, 7)	0.001

PVS, perivascular space; CMB, cerebral microbleed; DWMH, deep white matter hyperintensity; PWMH, periventricular white matter hyperintensity; FDMV, frontal deep medullary vein; PDMV, parietal deep medullary vein; ODMV, occipital deep medullary vein; TDMV, total deep medullary vein; CAA, cerebral amyloid angiopathy; HTNA, hypertensive arteriopathy; * = CAA-HTNA; ^†^ = HTNA-MIXED; ^#^ = CAA-MIXED; and *, ^†^, and ^#^ indicated comparison between the two groups and *p*-value < 0.05.

**Table 2:** Clinical and imaging characteristics and comparisons among study patients according to the DMV score.

Clinical and Imaging Characters	DMV Score			*p*-Value
	Mild (0–3) (*n* = 23)	Moderate (4–6) (*n* = 143)	Severe (7–9) (*n* = 66)	
Age (years), mean (SD)	48.08 ± 13.07 *	60.21 ± 13.10 ^†^	67.83 ± 11.68	<0.001
Male, *n*(%)	16(69.6)	95(66.4)	42(63.6)	0.858
Hypertension, *n*(%)	12(52.2) *	110(76.9)	54(81.8) ^#^	<0.001
Alcohol use, *n*(%)	5(21.7)	52(36.4)	19(28.8)	0.275
Tobacco use, *n*(%)	8(34.8)	59(41.2)	26(39.4)	0.833
Diabetes, *n*(%)	5(21.7)	34(23.8)	16(24.2)	0.97
Hyperlipidemia, *n*(%)	1(4.3)	34(23.8)	6(9.1)	0.07
Coronary heart disease, *n*(%)	1(4.3)	3(2.1)	4(6.1)	0.263
Stroke history, *n*(%)	0(0)	10(7.0)	10(15.2) **	0.045
PVS, median (IQR)	1(1, 2)	2(1, 2)	2(1, 2) ^#^	<0.05
CMB, median (IQR)	0(0, 1)	1(0,2)	1(0, 2) ^#^	<0.05
Lacuna, *n*(%)	14(60.9)	71(49.7)	27(40.9)	0.223
DWMH, median (IQR)	0(0, 2) *	2(1, 2) ^†^	2(1, 3) ^#^	<0.001
PWMH, median (IQR)	1(0, 2)	2(1, 2) ^†^	2(1, 3) ^#^	<0.001
CSVD score, median (IQR)	1(0, 3) *	3(1, 5) ^†^	3(2, 5) ^#^	<0.001

DMV, deep medullary vein; PVS, perivascular space; CMB, cerebral microbleed; DWMH, deep white matter hyperintensity; PWMH, periventricular white matter hyperintensity; * = mild-moderate; ^†^ = moderate-severe; ^#^ = mild-severe; *, ^†^, and ^#^ indicated comparison between the two groups and *p*-value < 0.05; and **, in mild-severe groups, *p*-value > 0.05 after adjustment.

**Table 3:** Correlations between CSVD markers and DMV score among study participants.

	TDMV Score	Age	Hypertension	Stroke History	PVS	CMB	Lacuna	DWMH	PWMH	CSVD Score
TDMV score	-									
Age	0.480 ^#^ (*p* < 0.001)	-								
Hypertension	0.202 * (*p* = 0.002)	0.181 * (*p* = 0.006)	-							
Stroke History	0.194 * (*p* = 0.003)	0.126 (*p* = 0.056)	0.030 (*p* = 0.653)	-						
PVS	0.190 * (*p* = 0.004)	0.211 * (*p* = 0.001)	0.016 (*p* = 0.804)	0.002 (*p* = 0.972)	-					
CMB	0.211 * (*p* = 0.001)	0.271 (*p* < 0.001)	0.177 * (*p* = 0.007)	0.197 * (*p* = 0.003)	0.246 ^#^ (*p* < 0.001)	-				
Lacuna	0.125 (*p* = 0.058)	0.264 (*p* < 0.001)	0.241 (*p* < 0.001)	0.143 * (*p* = 0.029)	0.286 ^#^ (*p* < 0.001)	0.417 ^#^ (*p* < 0.001)	-			
DWMH	0.310 ^#^ (*p* < 0.001)	0.501 ^#^ (*p* < 0.001)	0.188 * (*p* = 0.004)	0.200 * (*p* = 0.002)	0.278 ^#^ (*p* < 0.001)	0.516 ^#^ (*p* < 0.001)	0.558 ^#^ (*p* < 0.001)	-		
PWMH	0.295 ^#^ (*p* < 0.001)	0.528 ^#^ (*p* < 0.001)	0.243 ^#^ (*p* < 0.001)	0.279 ^#^ (*p* < 0.001)	0.293 ^#^ (*p* < 0.001)	0.496 ^#^ (*p* < 0.001)	0.559 ^#^ (*p* < 0.001)	0.830 ^#^ (*p* < 0.001)	-	
CSVD Score	0.308 ^#^ (*p* < 0.001)	0.424 ^#^ (*p* < 0.001)	0.243 ^#^ (*p* < 0.001)	0.224 * (*p* = 0.001)	0.468 ^#^ (*p* < 0.001)	0.793 ^#^ (*p* < 0.001)	0.717 ^#^ (*p* < 0.001)	0.811 ^#^ (*p* < 0.001)	0.790 ^#^ (*p* < 0.001)	-

TDMV, total deep medullary vein; PVS, perivascular space; CMB, cerebral microbleed; DWMH, deep white matter hyperintensity; and PWMH, periventricular white matter hyperintensity. Cells present correlations between variables of interest as correlation coefficients. Significance testing results (*p* value) reported in parentheses. *, *p* < 0.05; and ^#^, *p* < 0.001.

**Table 4:** ROC curve analysis of CSVD parameters among DMV groups.

Groups	Parameter	Cut-off	AUC	Sensitivity	Specificity
Mild vs. Moderate	Age	52.5	0.734	90.4	46.7
	PVS	1.5	0.630	62.4	57.9
	CMB	0.5	0.633	77.6	37.4
	DWMH	1.5	0.678	70.4	100
	PWMH	1.5	0.630	65.6	51.4
	CSVD score	1.5	0.695	83.2	38.3
Mild vs. Severe	Age	54.5	0.879	67.8	78.3
	PVS	2.5	0.697	22.4	100
	CMB	0.5	0.695	69.9	52.2
	DWMH	0.5	0.789	80.4	52.2
	PWMH	1.5	0.747	52.4	69.6
	CSVD score	1.5	0.793	72.7	60.9
Moderate vs. Severe	Age	54.5	0.668	86.7	78.3
	DWMH	0.5	0.621	97.0	52.2
	PWMH	1.5	0.628	71.2	69.6
	CSVD score	1.5	0.617	86.4	60.9

DMV, deep medullary vein; PVS, perivascular space; CMB, cerebral microbleed; DWMH, deep white matter hyperintensity; and PWMH, periventricular white matter hyperintensity.

**Table 5:** Multivariate analysis for the ICH subtypes.

		OR	OR Value (95% CI)		*p*-Value
			Lowest	Highest	
CAA-related vs. Mixed ICH	age	1.074	1.028	1.122	*p* = 0.001
	hypertension	5.169	1.969	13.567	*p* = 0.001
HTNA-related vs. Mixed ICH	DWMH	0.452	0.253	0.809	*p <* 0.05
	PVS	0.633	0.416	0.963	*p <* 0.05

PVS, perivascular space; DWMH, deep white matter hyperintensity; and CI, Confidence interval.

## Data Availability

The data used in this study are not publicly available due to restrictions in the data-sharing agreement.

## Supplementary Materials

The following supporting information can be downloaded at: https://www.mdpi.com/article/10.3390/brainsci13091315/s1, Table S1: Magnetic resonance imaging protocols; Table S2: The variability in all the MRI scores.

## Abbreviations

CSVD Cerebral small vascular disease, ICH intracerebral hemorrhage, DMV deep medullary vein, FDMV frontal deep medullary vein; PDMV parietal deep medullary vein; ODMV occipital deep medullary vein; TDMV total deep medullary vein; WMH white matter hyperintensity, DWMH deep white matter hyperintensity; PWMH periventricular white matter hyperintensity, CMB cerebral microbleed, PVS perivascular space, BG basal ganglia, HTNA hypertensive arteriopathy, CAA cerebral amyloid angiopathy, SD standard deviations, IQRs interquartile ranges, ROC receiver operating characteristic, BBB blood-brain barrier, MRI magnetic resonance imaging, SWI Susceptibility-weighted imaging, CT computer tomography, ICC intraclass correlation efficient.
