# Supplementary material for: Deep Medullary Vein and MRI Markers Were Related to Cerebral Hemorrhage Subtypes
Source: Brain Sci. 2023 Sep 13;13(9):1315. doi: 10.3390/brainsci13091315 (PMC10526710; doi:10.3390/brainsci13091315)
Supplement: Supplementary file 1 [file brainsci-13-01315-s001.zip › brainsci-2539386-supplementary.pdf]

**Table S1** Magnetic resonance imaging protocols		
United Imaging	Sequences	Parameters
	T1WI	TR = 4500 ms; TE = 102.5 ms; FOV = 240 mm × 200 mm; flip angle = 90°; slice thickness = 6 mm, matrix number = 352 × 316
	T2WI	TR = 6.4 ms; TE = 2.5 ms; inversion time(TI) = 830 ms; FOV = 250 mm × 220 mm; flip angle = 8°; slice thickness = 1 mm, matrix number = 240 × 240
	T2-Flair	TR = 8500 ms; TE = 123 ms; inversion time(TI) = 2500 ms; FOV = 230 mm × 200 mm; flip angle = 90°; slice thickness = 6 mm, matrix number = 320 × 240
Siemens	T1WI	TR = 2000 ms; TE = 2.48 ms; FOV = 240 mm × 240 mm; flip angle = 90°; slice thickness =1 mm, matrix number = 352 × 316
	T2WI	TR = 2880 ms; TE = 119 ms; FOV = 240 mm × 240 mm; flip angle = 90°; slice thickness = 5 mm, matrix number = 512 × 512
	T2-Flair	TR = 6877 ms; TE = 146 ms; FOV = 240 mm × 240 mm; flip angle = 90°; slice thickness = 5 mm, matrix number = 512 × 512
GE	T1WI	TR = 2050 ms; TE = 8.7 ms;inversion time(TI) = 720 ms; FOV = 240 mm × 240 mm; flip angle = 90°; slice thickness =5 mm, matrix number = 320 × 196
	T2WI	TR = 4300 ms; TE = 106 ms; FOV = 240 mm × 240 mm; flip angle = 90°; slice thickness = 5 mm, matrix number = 240 × 240
	T2-Flair	TR = 7600 ms; TE = 148 ms; inversion time(TI) = 1900 ms; FOV = 240 mm × 240 mm; flip angle = 90°; slice thickness = 5 mm, matrix number = 288 ×192
TR, repetition time; TE, echo time; FOV, field of view; Flair, fluidattenuated inversion recovery		

Table S2 The variability in all the MRI scores

	ICC	*P*-value
FDMV	0.830	<0.001
PDMV	0.748	<0.001
ODMV	0.875	<0.001
TDMV	0.906	<0.001
PVS	0.777	<0.001
Lacuna	0.932	<0.001
CMB	0.987	<0.001
DWMH	0.946	<0.001
PWMH	0.936	<0.001
TWMH	0.958	<0.001
CSVD score	0.953	<0.001

FDMV, frontal deep medullary vein; PDMV, parietal deep medullary vein; ODMV, occipital deep medullary vein; TDMV, total deep medullary vein; PVS, perivascular space; CMB, cerebral microbleed; DWMH, deep white matter hyperintensity; PWMH, periventricular white matter hyperintensity; TWMH, total white matter hyperintensity; CSVD,cerebral small vessel disease; ICC,intraclass correlation efficient;
